# The Multifaceted Role of Epoxide Hydrolases in Human Health and Disease

**DOI:** 10.3390/ijms22010013

**Published:** 2020-12-22

**Authors:** Jérémie Gautheron, Isabelle Jéru

**Affiliations:** 1 Centre de Recherche Saint-Antoine (CRSA), Inserm UMRS_938, Sorbonne Université, 75012 Paris, France; jeremie.gautheron@inserm.fr; 2 Institute of Cardiometabolism and Nutrition (ICAN), Hôpital Pitié-Salpêtrière, Assistance Publique-Hôpitaux de Paris, 75013 Paris, France; 3 Laboratoire Commun de Biologie et Génétique Moléculaires, Hôpital Saint-Antoine, Assistance Publique-Hôpitaux de Paris, 75012 Paris, France

**Keywords:** epoxide hydrolase, EPHX, mEH, sEH, fatty acids, genetics

## Abstract

Epoxide hydrolases (EHs) are key enzymes involved in the detoxification of xenobiotics and biotransformation of endogenous epoxides. They catalyze the hydrolysis of highly reactive epoxides to less reactive diols. EHs thereby orchestrate crucial signaling pathways for cell homeostasis. The EH family comprises 5 proteins and 2 candidate members, for which the corresponding genes are not yet identified. Although the first EHs were identified more than 30 years ago, the full spectrum of their substrates and associated biological functions remain partly unknown. The two best-known EHs are EPHX1 and EPHX2. Their wide expression pattern and multiple functions led to the development of specific inhibitors. This review summarizes the most important points regarding the current knowledge on this protein family and highlights the particularities of each EH. These different enzymes can be distinguished by their expression pattern, spectrum of associated substrates, sub-cellular localization, and enzymatic characteristics. We also reevaluated the pathogenicity of previously reported variants in genes that encode EHs and are involved in multiple disorders, in light of large datasets that were made available due to the broad development of next generation sequencing. Although association studies underline the pleiotropic and crucial role of EHs, no data on high-effect variants are confirmed to date.

## 1. Introduction

Epoxide hydrolases (EHs) constitute a small protein family, first characterized as a group of detoxifying enzymes [1]. EHs catalyze the hydrolysis of epoxides to their corresponding dihydrodiols, using activated water [2]. Epoxides are three-membered cyclic ethers with a high reactivity and electrophilicity, due to their strained cyclic structure and the polarization of electrons on the C-O bond. Epoxides are involved in the development of cancers and organ damages through interaction with DNA, lipids, and proteins [3]. Therefore, it is of vital importance for the organism to regulate levels of these reactive species. Epoxides are the primary metabolites of xenobiotic-metabolizing enzymes, especially cytochrome P450. These xenobiotics include drugs, chemicals, environmental pollutants, and toxins. EHs, which rapidly catalyze the hydrolysis of epoxides to more soluble and easily excretable metabolites, play a key role in the detoxification of these reactive epoxides. Other epoxides arise from oxidative metabolism of endogenous compounds. As an example, epoxides from fatty acids were shown to mediate signaling pathways and an alteration in their level induces many disorders. In this regard, EHs regulate numerous physiological functions in controlling tissue levels of these biologically active mediators.

Phylogenetic studies of epoxide hydrolases reveal that they are present in multiple organisms, including animals, insects, plants, fungi, and bacteria [4]. There are at least seven members within the epoxide hydrolase family in humans [5,6]. The cDNA for human EPHX1 was isolated in 1988 [7], and that of EPHX2 in 1993 [8]. This review mostly focuses on these two epoxide hydrolases, which have a wide expression pattern and numerous substrates. Two additional epoxide hydrolases, EPHX3 and EPHX4, were identified more recently [5]. Data related to these newly discovered EHs remain relatively scarce. The other three potential EHs, which are the cholesterol, hepoxilin, and leukotriene A4 epoxide hydrolases, have narrower substrate specificity, and their characteristics remain partly elusive.

This review summarizes the current knowledge on this protein family, while pointing out the particularities of each EH. It also presents an updated and critical view of genetic studies related to this class of enzymes.

## 2. Materials and Methods

We used NCBI PubMed and ScienceDirect databases for literature research. Publications were searched using the terms—epoxyde hydrolase; EPHX; mEH; sEH; LTA4H; hepoxilin; metabolism; detoxification; xenobiotics; variants; and mutations. Only publications in English were included. Articles were screened to identify potentially relevant studies. Reference lists of retrieved literature were also searched for relevant publications. Several databases were used to present an updated view on genetic studies related to EHs: orphanet (https://www.orpha.net/consor/cgi-bin/index.php?lng=EN); dbSNP (https://www.ncbi.nlm.nih.gov/snp/); gnomAD (https://gnomad.broadinstitute.org/); and the gene page from NCBI (https://www.ncbi.nlm.nih.gov/gene/). The determination of protein sequence identity between EHs was performed using the BLAST tool from NCBI (https://blast.ncbi.nlm.nih.gov/Blast.cgi).

## 3. EPHX1: Gene, Structure, and Enzymatic Functions

### 3.1. From Gene to Protein

The human *EPHX1* gene is located on chromosome 1, consists of 9 coding exons, and encodes a protein of 455 amino acids [9]. The EPHX1 protein, also known as microsomal epoxide hydrolase (mEH), is highly conserved throughout evolution [10]. Although the three-dimensional (3D) structure of EPHX1 is still not available, the quaternary structure of a closely homologous enzyme was determined from the fungus *Aspergillus niger* [11].

In mammals, the gene is widely expressed with a predominant expression in liver, adrenal gland, ovary, and adipose tissue [12]. The tissue-specific expression of human *EPHX1* depends on alternative promoter elements [13,14], and is modulated by numerous factors. It is directly regulated by the hormonal system. In this regard, Kim et al. showed that EPHX1 protein levels are positively and negatively regulated by insulin and glucagon, respectively, in cultured primary rat hepatocytes [15]. In addition, Popp et al. showed that progesterone positively influences the levels of EPHX1 during the menstrual cycle, in the endometrium [16]. Finally, numerous bioactive compounds including proliferator-activated receptor (PPAR) agonists, heavy metals, barbiturate (e.g., phenobarbitone) and carcinogenic derivative (e.g., *N*-acetylaminofluorene) induce the expression of *EPHX1* through the regulation of various transcription factors, such as Nrf2 and GATA-4 [17,18,19]. EPHX1 inhibitors include growth hormone, lipopolysaccharide, and glucocorticoids, among others.

EPHX1 is localized mainly in the microsomal fraction of the endoplasmic reticulum [12]. The protein is retained in microsomal membranes by a single transmembrane segment, which is located at the N-terminus and comprises around 20 amino acids [20]. The C-terminal part of the protein is exposed at the cytosolic membrane surface and comprises the catalytic domain [21]. Alternatively, EPHX1 is also found to be associated with the plasma membrane and particularly on the sinusoidal plasma membrane of hepatocytes [22].

Taken together, the wide and tightly controlled expression of *EPHX1* suggests that it exerts pleiotropic functions in multiple tissues with potential consequences in the pathophysiology of numerous conditions.

### 3.2. Mechanisms of Action and Substrates

**Figure 1:**
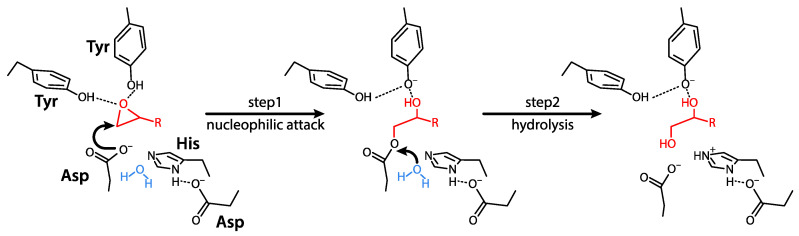
Two-step enzymatic mechanism of α/β hydrolase fold EHs. In the first step, EH forms a covalent bond with its substrate. In the second step, the resulting ester is hydrolyzed. The epoxide core of the substrate is depicted in red, water (H_2_O) in blue, and the different parts of the EH enzyme in black, with the catalytic site residues appearing in bold characters. This figure is adapted from Decker et al. [10].

EPHX1 belongs to the superfamily of α/β hydrolase fold enzymes. EPHX1 is responsible for epoxide reactive species biotransformation and thereby plays a key role in cell metabolism and homeostasis. The mechanism of hydrolysis of epoxides to dihydrodiols by EPHX1 involves two chemical steps. A fast-nucleophilic attack leads to the formation of an enzyme-substrate ester intermediate. Thereafter, hydrolysis of this complex to dihydrodiols through activated water is mediated by a charge relay system [23] (Figure 1).

EPHX1 substrates include xenobiotics, as well as endogenous molecules. Previous reports detailed the structural formulas of these substrates [24,25]. EPHX1 is considered to be the main EH responsible for the metabolism of xenobiotic epoxides. Examples for environmental contaminants metabolized by EPHX1 include epoxide derivatives of polycyclic aromatic hydrocarbons and alkenic-based substrates, such as styrene oxide. Several anticonvulsant drugs are well-known EPHX1 substrates, including carbamazepine and phenytoin [24]. Endogenous substrates include epoxides of fatty acids, which are important regulatory lipid mediators. These epoxy fatty acids comprise epoxyeicosatrienoic acids (EETs) and epoxy-octadecenoic acids (EpOMEs), which are formed by CYP epoxygenases from arachidonic acid (AA) and linoleic acid (LA), respectively [26,27,28]. EPHX1 catalyzes their hydrolysis to their respective diols, the so-called dihydroxyeicosatrienoic acids (DHETs) and dihydroxyoctadecanoic acids (DiHOMEs). EETs display several beneficial effects, including anti-inflammation, anti-platelet aggregation, vasodilatation, and anti-fibrotic properties [29]. Several epoxysteroids were also found to be EPHX1 substrates, such as androstene oxide (16α,17α-epoxyandrosten-3-one) and estroxide (epoxyestratrienol) [30]. The full spectrum of EPHX1 substrates might be difficult to establish due to the short half-life of epoxide intermediates, which are therefore difficult to measure.

### 3.3. Physiological Functions

**Figure 2:**
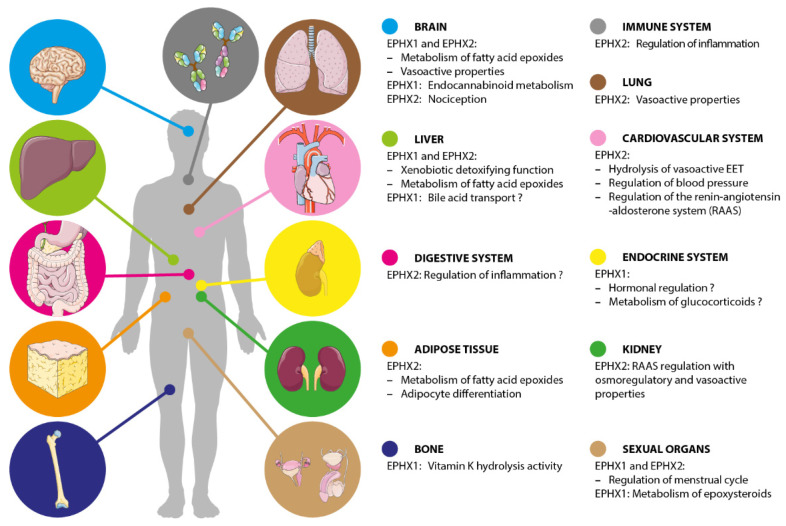
Kaleidoscopic view of the main functions of EPHX1 and EPHX2. Some biological functions remain hypothetical as the associated findings are not yet replicated or need further investigations.

The function of EPHX1 was discovered and deduced by a number of complementary approaches—the detailed study of its expression profile, mice models, genetic association studies in multiple disorders, and pharmacological inhibition studies. Although all these strategies help to build our global vision of the function of this pleiotropic enzyme, some data are uncertain or remain to be confirmed in humans. The following paragraph and associated figure (Figure 2) cannot be exhaustive of all previously published studies but provide a general overview of the current knowledge on EPHX1 functions.

EPHX1 was first purified from rabbit liver [31] and then characterized in human liver [32], suggesting a prominent role in the physiology of this organ. In hepatocytes, the primary function of EPHX1 is to catalyze the conversion of a broad spectrum of xenobiotic epoxide substrates, derived from drugs, toxic, and procarcinogen compounds, to more polar diol metabolites. This detoxification function due to the breakdown of xenobiotics, protects cells against chemical-induced genotoxic and cytotoxic stress [33]. However, it was also shown that EPHX1 bioactivates a number of potent carcinogens, by inducing the formation of reactive metabolites.

While EPHX1 mostly has a detoxifying function in liver, there is some evidence that it might play a major role in organ-specific physiological responses. First, EPHX1 regulates endogenous steroid metabolism by hydrolyzing several steroid epoxides, suggesting a functional role in energy metabolism and stress response (i.e., glucocorticoids), and sexual development and function (i.e., androgens and estrogens) [34]. In line, a decrease in estradiol formation was seen in human ovary, upon treatment with an EPHX1 inhibitor [35]. Second, it was proposed that EPHX1 might mediate bile acid transportation by regulating either the sodium-dependent transport of bile acids into hepatocytes [36], or the hepatocyte smooth endoplasmic reticulum (SER) vesicle system [37]. Third, EPHX1 is a major functional component of a multi-protein reductase complex that is responsible for vitamin K1 oxide reduction activity, suggesting that modulation of EPHX1 activity might contribute to blood coagulation and bone mineralization [38]. Finally, EPHX1 plays an important role in the endocannabinoid signaling pathway by metabolizing endocannabinoid 2-arachidonoylglycerol (2-AG) to free AA and glycerol [39]. Free AA, which can be further metabolized by cyclooxygenases, lipoxygenases, and cytochrome P450s to biologically active eicosanoids, are involved in inflammation, vasodilation, vasoconstriction, cell growth, and migration [40]. Thereby, EPHX1 might regulate stress, depression, as well as chronic pain, by modulating the endocannabinoid metabolism [41,42].

*EPHX1* knock-out mice are viable, fertile, normal in size, and do not display any gross physical or behavioral abnormalities [43]. Histological examination of several organs revealed no difference between *EPHX1* knock-out mice and their wildtype littermates. Mice carrying a gain-of-function variant, p.Glu404Asp, were also generated [44]. These mice did not present any obvious phenotype. However, they displayed an accelerated detoxification rate and faster metabolization of endogenous EETs in multiple organs, consistent with a broad impact of EPHX1 on endogenous EET metabolism [44].

EPHX1 is involved in cancer development and carcinogenesis. Knock-out mice are incapable of metabolizing the carcinogen 7,12-dimethylbenz[a]anthracene (DMBA), and therefore, are resistant to DMBA-induced skin cancer [43,45]. It suggests that EPHX1 by metabolizing pro-carcinogens such as polycyclic aromatic hydrocarbons (PAH), such as DMBA, might contribute to tumorigenesis [46]. Moreover, EPHX1 might also be involved in chemoresistance to cancers, such as acute myeloid leukemia, by metabolizing antitumor drugs [47]. The connection between EPHX1 and cancer development was further analyzed by correlating *EPHX1* expression in tumor tissues with disease progression and clinical outcomes. *EPHX1* expression was found to be high in 89% of tumor tissues in primary operable breast cancer [48], and was associated with poor disease outcome, notably in patients receiving tamoxifen treatment [49]. Furthermore, EPHX1 was differentially expressed in hepatocarcinoma and liver metastases, when compared to normal liver tissue, suggesting potential implications in tumor progression [50]. Finally, EPHX1 overexpression was observed in other human malignancies including lung, ovarian, and colorectal carcinomas [12]. Together, these findings indicate that variations in the hydrolysis activity of EPHX1 might influence cancer development.

### 3.4. EPHX1 Inhibitors

A number of EPHX1 inhibitors were developed to investigate the mechanistic aspects of epoxide hydrolysis in pathophysiology. These can be divided into two classes. The first class of inhibitors, including 1,1,1-trichloropropene-2,3-oxide and cyclohexene oxide, acts as substrates for EPHX1, through rapid binding but slowly dissociates, thereby preventing association of EPHX1 with its natural substrates [51]. The second class is carbamide-based EPHX1 inhibitors, including valpromide, which directly blocks the catalytic domain of EPHX1 [52]. Structural formulas for these inhibitors can be found in previous reports [24]. More potent amide-based EPHX1 inhibitors were also engineered, and inhibit EPHX1 activity by associating to the catalytic domain with low nanomolar dissociation [53,54]. EPHX1 inhibition was reported to be beneficial in the case of PAH exposure, and 1,1,1-trichloropropene-2,3-oxide reduced the carcinogenicity of DMBA [55]. Additionally, valpromide is an anti-convulsant drug, suggesting that EPHX1 might be targeted in epilepsy [52]. However, due to the detoxification properties of EPHX1, its inhibition might induce hepatotoxicity, and therefore, the use of EPHX1 inhibitors needs to be evaluated cautiously.

## 4. EPHX2: Gene, Structure, and Enzyme Function

### 4.1. From Gene to Protein

The EPHX2 gene, which is located on chromosome 8 and consists of 19 coding exons, encodes a protein of 555 residues. The EPHX2 protein also known as soluble epoxide hydrolase (sEH) is highly conserved throughout evolution. Nevertheless, the EPHX2 protein sequence is very different from that of EPHX1, with a protein sequence identity of 23%, as evaluated by a BLAST comparison in NCBI. *EPHX2* has a very wide expression pattern, the highest expression being detected in kidney, liver, and intestine [56]. Homocysteine and angiotensin-II were shown to induce *EPHX2* expression in human endothelial cells [57] and cardiomyocytes [58], respectively. *EPHX2* expression is also induced by peroxisome proliferators, including hypolipidemic drugs (e.g., fibrates and phtalates), through the regulation of PPAR [59]. EPHX2 is mainly present in the cell cytoplasm [60], and forms homodimers in solution. The N-terminal part of EPHX2 does not act as a membrane anchor, but contains a second catalytic site consisting of a phosphatase domain whose substrate remains unknown [61]. The C-terminal part of the protein provides the epoxide hydrolase activity [62].

### 4.2. Mechanisms of Action and Substrates

EPHX2 belongs to the superfamily of α/β hydrolase fold enzymes. The mechanism of epoxide hydration by EPHX2 is similar to that described for EPHX1 (Figure 1). EPHX2 3D structure is available and the binding of substrates and inhibitors to the enzymatic pocket was investigated by several teams [61,63]. EPHX2 displays a high affinity for epoxides of fatty acids [25,62,64]. Structural formulas for EPHX2 substrates were detailed in previous reports [25].

Among the best-known substrates of EPHX2 are the EETs, which come from AA and are metabolized in DHETs [29]. The discovery of EETs and associated metabolic pathways was made several decades ago. The appreciation of their key role in pathophysiological processes was greatly accelerated by the generation of EPHX2 inhibitors, the availability of commercial EETs, and the development of analytical methods for the quantification of EETs and their diol substrates in expert laboratories. EETs were shown to display beneficial effects in numerous conditions—inflammation, pain, hypertension, cardiac hypertrophy and arrhythmia, atherosclerosis, stroke, renal failure, blood clotting, chronic obstructive pulmonary disease (COPD), pulmonary hypertension, diabetes, and metabolic disease [63,65]. The positive impact of EPHX2 inhibition was demonstrated in various animal models, leading to the development of pharmacological EPHX2 inhibitors (see below).

Among other EPHX2 substrates, there are EpOMEs that come from LA and are metabolized to DiHOMEs. EPHX2 also takes part in the metabolism of omega 3-polyunsaturated fatty acids, such as eicosapentaenoic acid (EPA) and docosahexaenoic acid (DHA). EPA and DHA are primarily metabolized into the bioactive epoxyeicosatetraenoic acids (ETEs) and epoxydocosapentaenoic acids (DPEs), respectively [65]. Then, EPHX2 converts these epoxides to their corresponding 1,2-diols (i.e., DiHETEs and DiHDPEs). It was shown that these epoxides derived from EPA and DHA contribute to regulate blood pressure, inflammation, and metabolic disorders associated with obesity [66,67,68,69].

### 4.3. Physiological Functions

EPHX2 plays an important role in xenobiotic epoxide metabolism and shows complementary substrates specificity to EPHX1 [25]. However, the major physiological role of EPHX2 is the metabolism of epoxides derived from fatty acid, such as EETs and EpOMEs, also termed leukotoxins [29]. The following paragraphs also refer to Figure 2 and provide a general overview of the current knowledge on EPHX2 functions.

Arachidonic acid is metabolized through different pathways, including the cyclooxygenase and lipoxygenase pathways that produce prostaglandins and leukotrienes, respectively. These metabolites have pro-inflammatory properties and are currently targeted to reduce inflammation and pain [70]. More recently, a third pathway emerged, which is dependent on cytochrome P450s, and produces EETs and other epoxy fatty acids. These metabolites are bioactive, and in contrast to prostaglandins and leukotrienes, they efficiently regulate inflammation and pain by exhibiting anti-inflammatory properties [63]. However, EETs are rapidly converted into DHETs in the presence of EPHX2, losing their abilities to control inflammation and moving towards pro-inflammatory properties [71].

Linoleic acid (LA), an essential fatty acid, is epoxidized in vivo to EpOMEs. This epoxidation can be spontaneous with reactive oxygen species (ROS) or mediated by various cytochrome enzymes, such as P450s and cytochrome c [72]. In contrast to EETs, EpOMEs are cytotoxic, and inhibit the mitochondrial respiration by uncoupling oxidative phosphorylation. This leads to multiple organ failure, such as cardiotoxicity, renal failure, and acute respiratory distress syndrome (ARDS) [73,74]. However, the cytotoxic effect of EpOMEs is less potent than their DiHOMEs generated by EPHX2. Indeed, DiHOMEs trigger pronounced mitochondrial structural abnormalities by inhibiting the sodium–potassium pump and the electron transport chain itself, and by promoting the nuclear factor kappa B (NF-κB). EPHX2 inhibitors were then developed to prevent the conversion of EETs and EpOMEs. This consequently enhanced the pool of EETs with anti-inflammatory properties, while preserving the conversion of EpOMEs to DiHOMEs that are more potent inductors of the inflammatory processes.

EPHX2, with its broad expression and numerous substrates, was shown to act on vasculature, heart, kidney, liver, brain, lung, adipose tissue, inflammatory, and reproductive systems [71]. Consistently, it was shown to be implicated in numerous pathophysiological processes. Among them, we can list acute nociception, inflammation, and sepsis, inflammatory bowel disease, diabetes, pulmonary hypertension, ischemic injury in the heart and brain, as well as tumorigenesis. Due to this central physiological role, EPHX2 was deeply investigated as a therapeutic target.

### 4.4. EPHX2 Inhibitors

Similar to EPHX1 inhibitors, the first-generation of EPHX2 inhibitors work as competitive reversible drugs through rapid binding to EPHX2, with a slow dissociation [75]. Later, several other compounds derived from urea, amide, and carbamate were engineered, and shown to be potent inhibitors of EPHX2 activity, with low nanomolar dissociation [76]. Structural formulas for these inhibitors can be found in previous reports [65]. The effects of EPHX2 inhibition on inflammation are multiple. It decreases bone loss by modulating host inflammatory response in an inflammatory bone resorption experimental model, similar to what occurs in arthritis [77]. EPHX2 inhibition also ameliorates hyperalgesia, edema, and decreases the expression of important pro-inflammatory cytokines of Th1 and Th17 profiles, while increasing Treg cells in an experimental model of arthritis [78]. Furthermore, EPHX2 inhibition reduces inflammation in an experimental inflammatory bowel disease [79], increases the survival rate of mice after lipopolysaccharide-induced acute systemic inflammation [80], delays onset of nephritis, and ameliorates kidney damage in experimental model of lupus [81], and reduces renal inflammation and keratopathy in diabetic mice [82,83]. Altogether, these findings indicate that EPHX2 inhibitors represent a novel way to relieve systemic inflammation and is a new therapeutical approach in diseases with an inflammatory component.

EPHX2 inhibition also has beneficial effects on the cardiovascular system. These effects are essentially attributed to the increased pool of EETs, as a consequence of EPHX2 inhibition. EETs have potent vasodilator activities, by regulating the Ca^2+^-activated potassium channels on vascular smooth muscle cells [84]. They also regulate endothelial cell and vascular smooth muscle cell proliferation and migration, processes that are essential to vascular homeostasis [85]. Furthermore, EETs directly decrease NF-κB activation, and thereby, contribute to reducing inflammation during vascular damage and cardiovascular disease progression [86]. *Ephx2* knock-out mice were generated and male mice exhibited lower blood pressure, corroborating results seen with the chemical inhibition of EPHX2 [87]. Both sexes also display an altered AA metabolism, and formation of DHETs is significantly decreased [87].

Finally, it was shown that EPHX2 inhibition effectively alleviates inflammation and improves lung functions in experimental models of COPD and acute tobacco-induced inflammation [88,89,90]. The beneficial effects on lung were also attributed to increased levels of EETs. Altogether, inhibition of EPHX2 is a potential approach for enhancing the biological activity of EETs in cardiovascular and lung diseases.

Several clinical trials using EPHX2 inhibitors were initiated over the last decade [91]. They showed that pharmacological inhibition of EPHX2, notably with GSK2256294, is safe without serious adverse effects. Two clinical trials are still undergoing evaluation—the first one, referred as to NCT03486223, tests whether a given genetic variant in *EPHX2* (p.Arg287Gln) is associated with insulin sensitivity, and evaluates the effects of EPHX2 inhibition on type 2 diabetes; the second, referred as to NCT03318783, aims to evaluate the efficacy of EPHX2 inhibition in patients with aneurysmal subarachnoid hemorrhage [91].

## 5. EPHX3

Using a conserved 16-amino acid motif found in EH-related enzymes to query a human protein database, Decker et al. identified EPHX3 in 2012 [5]. EPHX3 is mostly expressed in the skin, lung, and gastrointestinal tract, which correspond to tissues that have contact surfaces with the outside. In contrast to EPHX1 and EPHX2, it is poorly expressed in the liver. The EPHX3 protein sequence displays few homologies with those of EPHX1 or EPHX2 (protein sequence identities of 22% and 29%, respectively), and shares the highest percentage of similarities with that of EPHX4 (protein sequence identity of 45%). EPHX3 is an endoplasmic reticulum resident protein with an N-terminal transmembrane anchor. It catalyzes the hydrolysis of several EETs, EpOMEs, and leukotoxin, whereas it is essentially inactive toward the generic EH substrate styrene oxide [5,92]. This suggests that EPHX3 is implicated in the metabolism of signaling molecules rather than in detoxification of xenobiotics. Intriguingly, the hydrolysis of these fatty acid epoxides did not seem to be altered in *Ephx3* knock-out mice [92]. Another recent study reported that EPHX3 plays a role in the formation of the water permeability barrier in the epidermis by catalyzing the hydrolysis of epoxide intermediates from the lipoxygenase pathway into linoleate triols [93]. EPHX3 is sensitive to inhibition by a subset of urea derivatives, initially developed as selective EPHX2 inhibitors and interfering with EET metabolism.

## 6. EPHX4 and Other Epoxide Hydrolases

EPHX4 was identified in 2012, together with EPHX3, by Decker et al. [5]. EPHX4 is mainly expressed within the brain. The deduced 362-amino acid protein has a predicted N-terminal transmembrane anchor, and the remainder of the molecule forms an epoxide hydrolase alpha/beta fold domain, with a central lid region. Its substrates and functions remain to be determined.

There are three additional EHs that were reported in humans. They do not seem to play an important role in xenobiotic metabolism, and their substrates are limited to a number of endogenous epoxides. The existence of a cholesterol epoxide hydrolase and a hepoxilin epoxide hydrolase was reported. However, there are few data on these enzymes and the corresponding genes are not identified to date. Leukotriene A4 hydrolase is encoded by the *LTA4H* gene. It has a wide expression pattern, though predominant in the blood and lung. This enzyme contains both hydrolase and aminopeptidase activities. Its protein sequence displays no significant similarities with those of EPHX proteins. LTA4H hydrolyzes leukotriene A4. The hydrolase activity is used in the final step of the biosynthesis of leukotriene B4, a chemotactic agent acting as a proinflammatory mediator. The aminopeptidase activity was shown to degrade proline-glycine-proline (PGP), a neutrophil chemoattractant and biomarker for COPD.

## 7. Variants of Epoxide Hydrolases in Monogenic Disorders

**Figure 3:**
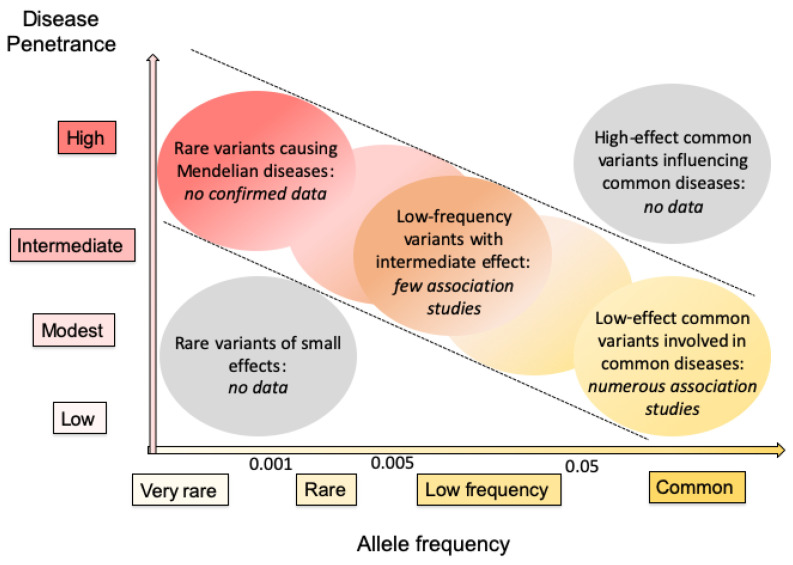
Current knowledge of variants in genes encoding human EHs based on allele frequency and disease penetrance. The disease penetrance is dependent on the strength of the variant genetic effect. Rare variants of small effects are hard to identify through genetic means, and are of low interest when investigating the pathophysiology of diseases. High-effect common variants influencing common diseases are very rare among human disorders. Consequently, most interest lies in genes shown within diagonal dotted lines. The data obtained to date for genes encoding human EHs are summarized in italics. This figure is based on concepts presented in two previous general reports on human genetics [95,96].

To date, there is no clear data involving any member of the EH family in Mendelian disorders (Figure 3). A patient with hypercholanemia and carrying two variants in *EPHX1*, one located in the promoter and the other in intron 1, was described in a previous report [94]. This patient presented elevated serum bile salt levels in the absence of observable hepatocellular injury, suggesting a defect in bile acid uptake. In this individual, the expression of *EPHX1* was reduced at the transcriptional and protein levels, and the authors suggested that the *EPHX1* variants might be responsible for the disorder. However, these data were never reproduced for more than 15 years, so the implication of *EPHX1* in hypercholanemia remains uncertain.

When investigating the potential pathogenicity of variants in a given gene, we can look at the observed/expected (o/e) ratio of variants in public databases such as gnomAD, which gathers information from more than 140,000 individuals around the world. This o/e ratio is a continuous measure of how tolerant a gene is to a certain class of variations, especially loss-of-function variants. When a gene has a low o/e value, it is under strong selection for that class of variation. For the interpretation of Mendelian disease cases, it is recommended to use the upper bound of the o/e CI < 0.35 as a threshold. For loss of-function-variants in EH genes, the o/e ratio are the following: *EPHX1*—0.74; *EPHX2*—0.94; *EPHX3*—1.16; *EPHX4*—0.55; and *LTA4H*—0.71. This shows that loss-of-function variants are present in genes from the EH family in the general population and that the rate of these variants is not predicted to be unexpected.

## 8. Variants of Epoxide Hydrolases as Susceptibility Factors to Human Diseases

Hundreds of variants of *EPHX1* and *EPHX2* were identified in the general population and numerous association studies were performed to try to implicate some of these variants in pathogenic processes (Figure 3). Mostly low-effect common variants associated with common disorders were identified and their real implication remains to be confirmed in many instances.

Two single nucleotide polymorphisms (SNPs) corresponding two missense variants were said to affect EPHX1 activity: c.337T>C—p.Tyr113His, and c.416A>G—p.His139Arg (NM_000120.4) [24]. Several dozen reports took interest in the p.Tyr113His variant. It was said to be a susceptibility factor to several conditions—alcohol dependence, liver cirrhosis, Crohn’s disease, chronic obstructive pulmonary disease, diabetes mellitus, preeclampsia, esophageal carcinoma, gastric cancer, breast cancer, lymphoblastic leukemia, ovarian cancer, and prostate cancer. It was also said to be a protective factor against several previously mentioned disorders, such as breast cancer and ovarian cancer, so that its real implication was not obvious. The second p.His139Arg variant was studied in similar association studies [24]. It was said to be a susceptibility factor to conditions such as isolated orofacial clefts, preeclampsia, esophageal carcinoma, and gastric cancer, though its implication in some of these disorders was also debated. A summary table presenting the link between these different variants and multiple diseases was proposed in a past review [24]. A few meta-analyses were performed to try to provide more statistical power to these association studies. Now that genetic data from next-generation sequencing are available in huge cohorts of individuals, these two variants are likely to be reclassified as polymorphisms or benign variants. Indeed, the gnomAD database, shows that the allele frequency of p.Tyr113His is 31.6% in the general population, with a frequency of homozygotes of 10.5%. In the same line, the allele frequency of p.His139Arg is 19.1%, with a frequency of 4.2% of homozygotes. Although this might depend on ethnicity and associated genetic background, these very high allele frequencies observed in healthy individuals rather argue against a major role of these variants in the disorders in which they are implicated.

A study on epigenetic factors revealed that the methylation level of the *EPHX1* promoter was significantly lower in patients with polycystic ovary syndrome than in controls, consistent with a role of *EPHX1* in steroidogenesis [97].

There are fewer data related to the implication of *EPHX2* variants in human disorders. At least five SNPs were shown to regulate the enzyme activity, either positively or negatively, and have attracted more interest [98,99]. These variants are listed hereafter along with their frequency in the general population: c.164A>G—p.Lys55Arg (9%); c.307C>T—p.Arg103Cys (1%); c.461G>A—p.Cys154Tyr (0.3%); c.860G>A—p.Arg287Gln (10%); c.1206_1208dup—p.Arg403dup (4%) (NM_001979.6). The p.Arg287Gln variant is one of the most studied and is associated with a number of conditions. A study reported a modification of the phenotype of *LDLR*-related familial hypercholesterolemia, in patients carrying this variant [100]. It was also shown to be a susceptibility factor to coronary artery calcification in African–Americans but not in white subjects [101], as well as a risk factor for cardiovascular disease in diabetic patients [102]. A report described an association of this same variant with insulin resistance in type 2 diabetic patients [103]. The p.Lys55Arg variant was shown to be a susceptibility factor to coronary disease, especially in Caucasians [104]. The p.Arg103Cys variant, associated with an increase in EPHX2 activity, was related with increased cell death in cortical neurons after ischemic injury, whereas the p.Arg287Gln variant had an opposite effect [105]. Many of these studies need to be repeated with a higher number of patients to allow final conclusions. Notably, several additional SNPs located in non-coding regions of *EPHX2*, as well as in the 5′ and 3′ untranslated regions, are associated with several disorders including cardiac disorders, male infertility, and kidney function.

## 9. Discussion

The first EHs were identified more than 30 years ago. A huge load of work was carried out to characterize their substrates and to try to design specific inhibitors. EHs appear to have evolved as critical regulators within complex transduction pathways, since they catalyzed the hydrolysis of numerous bioactive epoxidized metabolites serving as signaling molecules in their host organisms. However, it remains a challenge to clearly define the contribution of each EH in human health and diseases.

The identification of monogenic disorders due to pathogenic variants in members of the EH family would be very useful to better understand the true functions of these enzymes in humans. Unfortunately, such molecular defects are not identified thus far. Association studies led to contradictory data on the implication of particular SNPs in numerous conditions and diseases. Consequently, besides underlining the idea that EPHX1 and EPHX2 stand at the crossroads of many critical pathways, the true impact of these association studies for patients and knowledge of EHs remains in our opinion limited.

The study of particularities characterizing each EH might provide some clues to better understand their specific role. First, EHs differ by their expression pattern. The two EHs with the broadest expression pattern are EPHX1 and EPHX2, a first clue arguing for their pleiotropic effect. Second, EHs differ by their protein structure and functional domains. While the whole EH family shares a well-known epoxide hydrolysis activity, the bifunctional role of EHs with two distinct enzymatic domains is less clear. In this regard, the role of EPHX2 phosphatase and LTA4 aminopeptidase activities remains to be fully elucidated. It is all the more important that each of these enzymes seems to constitute a therapeutic target for the treatment of inflammatory diseases.

Third, EHs partly differ by their substrate selectivity. For a long time, it was thought that EPHX1 and EPHX2 fulfilled complete distinct roles. On the one hand, EPHX1 was well recognized as one of the main enzymes detoxifying xenobiotic epoxides. Owning to the high chemical reactivity of these compounds, EPHX1 was considered to be protective against mutagenic and carcinogenic initiation. Consequently, EPHX1 inhibition is thought to be rather deleterious, since it increases the toxicity of xenobiotics, as well as the risk of cancer and inflammatory diseases. On the other hand, EPHX2 was shown to orchestrate a variety of physiological functions, by mediating the formation of cytotoxic dihydrodiols at the expense of rather cytoprotective epoxides of fatty acids [24]. Consequently, EPHX2 inhibition emerged as a promising therapy to treat several diseases. However, the reality seems to be more complex. More recently, EPHX1 was shown to play a leading role in the hydrolysis of different fatty acid epoxides [26,27]. EPHX1 and EPHX2 indeed have partly redundant functions, with a significant overlap in substrate selectivity, and an identical catalytic triad to convert epoxides into diols. These overlapping functions are illustrated in a murine model of cardiac ischemia [26,27]. In this model, hypoxia induces cytochrome P450 epoxygenase-mediated production of AA-derived EETs, which have cardioprotective properties. By hydrolyzing EETs, EHs therefore reduce the beneficial effects of EETs [65]. Although previous in vitro studies showed that EETs are preferentially hydrolyzed by EPHX2, EPHX1 significantly contributes to EETs hydrolysis in vivo [26]. In this regard, ischemic hearts from *Ephx1 Ephx2* double knock-out mice, show a significantly better functional recovery due to the increased levels of EETs, than *Ephx2* single knock-out mice. These findings illustrate that in the absence of EPHX2, EPHX1 might take over the hydrolysis of EETs and consequently impair post-ischemic recovery [26,27]. Therefore, additional attention focused on the role of EPHX1 in lipid metabolism would certainly improve our understanding of critical signaling pathways. Similarly, efforts to elucidate the true function of EPHX1 in steroid and glucose metabolism would undoubtedly expand our understanding of this enzyme. Regarding the more recently identified EPHX3 and EPHX4, additional studies are needed to better understand the spectrum of their substrates and associated functions. The importance of the cholesterol epoxide hydrolase and hepoxilin hydrolase are suggested by the nature of their substrates, but our understanding also remains limited.

EHs also differ by their subcellular localization. The ER-resident EHs, including EPHX1 and EPHX3, have a kinetic advantage, since they can directly hydrolyze epoxides during their formation in the ER. In this regard, EPHX1, with its close physical proximity in the ER with CYP epoxygenases, acts with them in a coupled reaction [106], and hydrolyzes most of the epoxy fatty acids as they are formed under basal conditions [26]. When EET production rates are high (e.g., during ischemia or inflammation), EPHX1 capacity might be overwhelmed and EPHX2 would take the lead to hydrolyze most epoxy fatty acids. It was shown that EPHX1 is less abundant in some tissues, as compared to EPHX2, but it benefits from a more widespread expression [27]. EPHX1 was shown to be tens- to thousands-fold slower in in vitro epoxy fatty acid hydrolysis assays, as compared to EPHX2. In liver homogenates and microsomes, where cell compartmentalization is largely destroyed, epoxides might diffuse more easily and thus increase the probability of being subjected to EPHX2-mediated hydrolysis. This might explain why EPHX1 effects are mainly seen in vivo, but not in assays with homogenates or cellular fractions [27]. The major difference between EPHX1 and EPHX3, the two ER-resident EHs, is their Km for fatty acid epoxides. While both enzymes should afford a similar diol to epoxide ratio at low epoxide formation rates, this ratio would strongly decrease for EPHX1 when the substrate concentration increases, while the ratio would stay constant for EPHX3, due to its extremely high Km [5].

All these points help to synthesize our thoughts regarding the respective role of each EH. Nevertheless, several previous data also showed that their functions are partly interdependent. For example, loss of either EPHX1 or EPHX2 induces the transcriptional upregulation of the residual epoxyde hydrolase gene: *Ephx2* expression is significantly increased in liver from *Ephx1* knock-out mice and vice versa [27]. We also previously discussed the redundant complementary role of EPHX1, EPHX2, and EPHX3, depending on the amount of fatty acid epoxides to be metabolized. There is a consensus that the inhibition of EPHX1 is generally detrimental, and that the inhibition of EPHX2 is beneficial to numerous processes. However, considering the overlap in these enzyme substrates and physiological functions, we can guess that EPHX1 inhibition affects the metabolism of fatty acid epoxides and that EPHX2 inhibition impacts the metabolism of some xenobiotic reactive metabolites. Therefore, it is a key point to evaluate the selectivity of inhibitors towards either EPHX1 or EPHX2, as done previously for a number of candidates. Considering that some EPHX2 inhibitors also target EPHX3 [5,92], it also remains to be established whether EPHX2-selective inhibition or the mixed-type blockade of EET hydrolysis is more favorable in the treatments of disorders. Further investigations of EH-dependent metabolism are clearly warranted to answer these decisive questions on the physiological roles of human EHs in cell homeostasis.

## Abbreviations

2-AG	Endocannabinoid 2-arachidonoylglycerol
AA	Arachidonic acid
ARDS	Acute respiratory distress syndrome
COPD	Chronic obstructive pulmonary disease
DiHOME	Dihydroxyoctadecamoic acid
DHA	Docosahexaenoic acid
DHET	Dihydroxyeicosatrienoic acid
DiHDPE	Dihydroxydocosapentaenoic acid
DiHETE	Dihydroxyeicostetraenoic acid
DMBA	7,12-dimethylbenz[a]anthracene
DPE	Epoxydocosapentaenoic acid
EH	Epoxide hydrolase
EPA	Eicosapentanoic acid
EpOME	Epoxyoctadecenoic acid
ETE	Epoxyeicosatetraenoic acid
ETT	Epoxyeicosatrienoic acid
LA	Linoleic acid
mEH	Microsomal epoxide hydrolase
NF-κB	Nuclear Factor kappa B
PPAR	Proliferator-activated receptor
sEH	Soluble epoxide hydrolase
